# Local and Landscape Factors Determining Occurrence of Phyllostomid Bats in Tropical Secondary Forests

**DOI:** 10.1371/journal.pone.0035228

**Published:** 2012-04-18

**Authors:** Luis Daniel Avila-Cabadilla, Gerardo Arturo Sanchez-Azofeifa, Kathryn Elizabeth Stoner, Mariana Yolotl Alvarez-Añorve, Mauricio Quesada, Carlos Alonso Portillo-Quintero

**Affiliations:** 1 Centro de Investigaciones en Ecosistemas, Universidad Nacional Autónoma de México, Morelia, México; 2 Earth and Atmospheric Sciences Department, University of Alberta, Edmonton, Canada; 3 Department of Biological and Health Sciences, Texas A & M University, Kingsville, Texas, United States of America; 4 Centro de Estudios Botánicos y Agroforestales, Instituto Venezolano de Investigaciones Científicas, Maracaibo, Venezuela; University of Western Australia, Australia

## Abstract

Neotropical forests are being increasingly replaced by a mosaic of patches of different successional stages, agricultural fields and pasture lands. Consequently, the identification of factors shaping the performance of *taxa* in anthropogenic landscapes is gaining importance, especially for *taxa* playing critical roles in ecosystem functioning. As phyllostomid bats provide important ecological services through seed dispersal, pollination and control of animal populations, in this study we assessed the relationships between phyllostomid occurrence and the variation in local and landscape level habitat attributes caused by disturbance. We mist-netted phyllostomids in 12 sites representing 4 successional stages of a tropical dry forest (initial, early, intermediate and late). We also quantitatively characterized the habitat attributes at the local (vegetation structure complexity) and the landscape level (forest cover, area and diversity of patches). Two focal scales were considered for landscape characterization: 500 and 1000 m. During 142 sampling nights, we captured 606 individuals representing 15 species and 4 broad guilds. Variation in phyllostomid assemblages, ensembles and populations was associated with variation in local and landscape habitat attributes, and this association was scale-dependent. Specifically, we found a marked guild-specific response, where the abundance of nectarivores tended to be negatively associated with the mean area of dry forest patches, while the abundance of frugivores was positively associated with the percentage of riparian forest. These results are explained by the prevalence of chiropterophilic species in the dry forest and of chiropterochorous species in the riparian forest. Our results indicate that different vegetation classes, as well as a multi-spatial scale approach must be considered for evaluating bat response to variation in landscape attributes. Moreover, for the long-term conservation of phyllostomids in anthropogenic landscapes, we must realize that the management of the habitat at the landscape level is as important as the conservation of particular forest fragments.

## Introduction

Tropical landscapes have been increasingly modified by human activities, altering their natural structure and the course of ecological processes. In the Neotropics, cattle raising, agriculture and logging, have severely modified the natural vegetation, causing their replacement by a mosaic of patches of different successional stages, agricultural fields, and pasture lands [1], [2], 3. Under this scenario, considered by some authors the predominant habitat for wildlife in the near future [4], [5], the identification of factors that shape the *taxa* distribution and performance is gaining importance [6], [7]. Special attention must be paid to *taxa* playing critical roles in ecosystem functioning, which will help to maintain ecosystem structure and key ecological processes [8], [9].

Bats are considered an important component of biodiversity in the Neotropics, as well as a keystone group [10], [11], [12]. Due to their dramatic ecological and evolutionary radiation, they occupy virtually every trophic level, from primary to tertiary consumers, interacting with a large spectrum of organisms and regulating complex ecological processes [11], [13], [14], [15], [16]. They play an important role in ecosystem functioning by providing ecological services such as seed dispersal, pollination, and control of invertebrate and small vertebrate populations; they also contribute to the recycling and translocation of nutrients and energy in the ecosystem [11], [16], [17], [18], [19], [20], [21], [22], [23], [24]. In the Neotropics, bats visit and presumably pollinate approximately 573 species and disperse seeds from 549 species [25], [26], contributing to the maintaining of plant diversity, connecting distant plant populations via pollen and seed movement, and promoting forest regeneration in degraded lands via seed dispersal. In some Neotropical regions, nearly half of the most abundant pioneer plant species are bat-dispersed (i.e. *Solanum*, *Cecropia*, *Piper*, *Vismia*) [17].

The response of bats to anthropogenic disturbance in Neotropical regions has received increasing attention during the last twenty years but still remains poorly understood as studies have reported contradictory results (Table 1 in [27]). Some studies suggest that bats are more tolerant to habitat modification than other animals attributing this to: (1) their capacity to fly, crossing habitat boundaries and open areas (including physical barriers for other species), (2) their ability to exploit resources that are patchy in space and time, and (3) their capacity to shift diets or adapt their behavior to resource availability [13], [28], [29], [30], [31], [32], [33], [34], [35], [36]. Other studies, in contrast, suggest that bats are sensitive to habitat loss or modification and to the resulting variation in habitat structure, food and shelter. Bat responses to habitat alteration reported at the assemblage, ensemble, population and individuals levels include: (1) changes in species composition, (2) reductions in species diversity, (3) reductions in abundance, (4) strong deviations in sex ratios, (6) changes in their foraging patterns, and (5) the presence of more physiologically stressed individuals in fragments [8], [37], [38], [39], [40], [41], [42], [43], [44], 45,46,47,48,49,50,51,52,53. We use the terms assemblage and ensemble *sensu* Fauth et al. [54], who recognized as assemblage a phylogenetically bounded group of species inhabiting a given local habitat (i.e. phyllostomid assemblage) and as ensemble a set of species within an assemblage that use a similar set of resources (i.e. phyllostomid frugivores).

**Table 1:** Summary statistics of parameters at population, ensemble and assemblage-level.

Parameter	Mean	SD	Range
**Population-level**			
*Micronycteri microtis* (GI)	0.00	0.02	0–0.06
***Glossophaga soricina*** (N)	0.62	0.68	0–2.00
***Glossophaga commissarisi*** (N)	0.17	0.32	0–1.14
***Leptonycteris yerbabuenae*** (N)	0.20	0.37	0–1.07
*Choeroniscus godmani* (N)	0.00	0.02	0–0.06
*Musonycteris harrisoni* (N)	0.01	0.02	0–0.06
*Carollia sp.* (F)	0.03	0.11	0–0.39
***Artibeus jamaicensis*** (F)	1.26	1.72	0–6.22
*Artibeus watsoni* (F)	0.09	0.14	0–0.47
***Artibeus phaeotis*** (F)	0.14	0.23	0–0.72
***Artibeus lituratus*** (F)	0.24	0.36	0–1.28
*Sturnira lilium* (F)	0.07	0.12	0–0.29
*Centurio senex* (F)	0.01	0.03	0–0.11
*Chiroderma salvini* (F)	0.01	0.03	0–0.11
***Desmodus rotundus*** (S)	0.60	1.08	0–3.78
**Ensemble-level**			
S_8_N*	1.65	1.06	0–2.98
AbN	1.00	1.29	0–4.21
S_8_F*	2.65	1.18	1–5.14
AbF	2.52	3.15	0.38–14.22
**Assemblage-level**			
SC_1_	0	0.50	−0.99–0.58
SC_2_	0	0.55	−0.75–1.17
S_8_P*	4.61	1.97	1.00–8.04
AbP	3.46	3.89	0.38–14.22

Parameters at population-level: capture rate (individuals/night) as indicator of species local abundance. Parameters at ensemble-level: rarified number of nectarivorous (S_8_N) and frugivorous species (S_8_F), capture rate of nectarivores (AbN) and frugivores (AbF). Parameters at assemblage-level: scores of the first (SC_1_) and second (SC_2_) ordination axis reflecting assemblage's dissimilarities in species composition and structure, rarified number of phyllostomid species (S_8_P) and capture rate of phyllostomids (AbP). Mean: mean per site of the parameters at population, ensemble and assemblage-level. SD: standard deviation. Species in bold are those analyzed at the population-level. Species ensemble assignations are shown between parentheses: gleaning insectivores (GI), nectarivores (N), frugivores (F) and sanguivores (S). The number of sites sampled was 12 for all parameters except for three, which are marked with an asterisk. In these three parameters the site P1 was excluded from the analyses due to its low number of sampling nights.

A better understanding of bat response to habitat disturbance demands us to move from the dichotomous and qualitative description of habitat (i.e. fragmented vs. continuous forest, disturbed vs. undisturbed habitat) to a quantitative characterization of habitat attributes [55], [56]. Additionally, because the occurrence of vagile species in a particular fragment is greatly determined by the landscapes attributes, we need to evaluate the effect of variation not only on local habitat attributes (i.e. tree density, canopy cover, and fragment shape and area), but also on the spatial configuration and composition of the landscape at different focal scales [14], [32], [39], [46], [56], [57].

The main objective of this study was to identify potential explanatory relationships between changes in phyllostomid bat assemblages, ensembles and populations and the variation in habitat attributes, at local and landscape levels, caused by the two most common anthropogenic disturbances in the Neotropics: agriculture and cattle raising [1]. We focus on phyllostomid bats because this is the most diverse bat family in the Neotropics, in both taxonomic and functional terms, containing most of the foraging guilds and all the nectarivorous and frugivorous species [15]. The study was carried out in a tropical dry forest as this is one of the most widespread and disturbed neotropical systems [58], [59]. In order to adequately evaluate bat response to habitat disturbance we quantitatively characterized the habitat attributes that could influence bat occurrence at the local and landscape levels and at different focal scales. We finally discuss the implications of our results for the study and conservation of phyllostomid bats in anthropogenic landscapes.

To our knowledge, this is the first detailed investigation evaluating, at the landscape level, how variation in habitat attributes determines the occurrence of bats in an anthropogenic dry forest landscape. Our study is also one of the few in the Neotropics comparing the importance of local vs landscape level habitat attributes on bat presence and abundance [31], [46].

Based on results from previous studies we made the following predictions: (1) variation in phyllostomid assemblage composition, which includes a significant portion of species tightly associated with mature forest (6 of 15 species in the study region, [27]), will be mainly explained by variation in vegetation structural complexity and in vegetation cover; and (2) because of their contrasting ecological requirements (i.e. food resources), the abundance of nectarivores and frugivores will respond in a guild-specific way to changes in local and landscape habitat attributes [56]. Specifically, we expect to find the highest abundance of frugivores in sites surrounded by the highest amount of riparian vegetation, which hosts most of the chiropterochoric species of the region [60]. In contrast, the highest nectarivore abundance is expected to occur in sites surrounded by a reduced amount of dry forest, as the most abundant nectarivorous species in the region tend to occur in higher abundance in the early successional stages of the tropical dry forest [27].

## Materials and Methods

### Ethics Statement

Bat captures and handling were in accordance with the laws of the Mexican Government and with the authorization of the Oficina de Fauna Silvestre, Mexico (SGPA/DGVS Permit 3644 to KES). This study was also approved by the Secretaría de Medio Ambiente y Recursos Naturales (SEMARNAT), and the Consejo Nacional de Ciencia y Tecnología (CONACYT) from Mexico (Projects 2002-C01-0597 and CB-2005-51043).

### Study area and sampling sites

**Figure 1:**
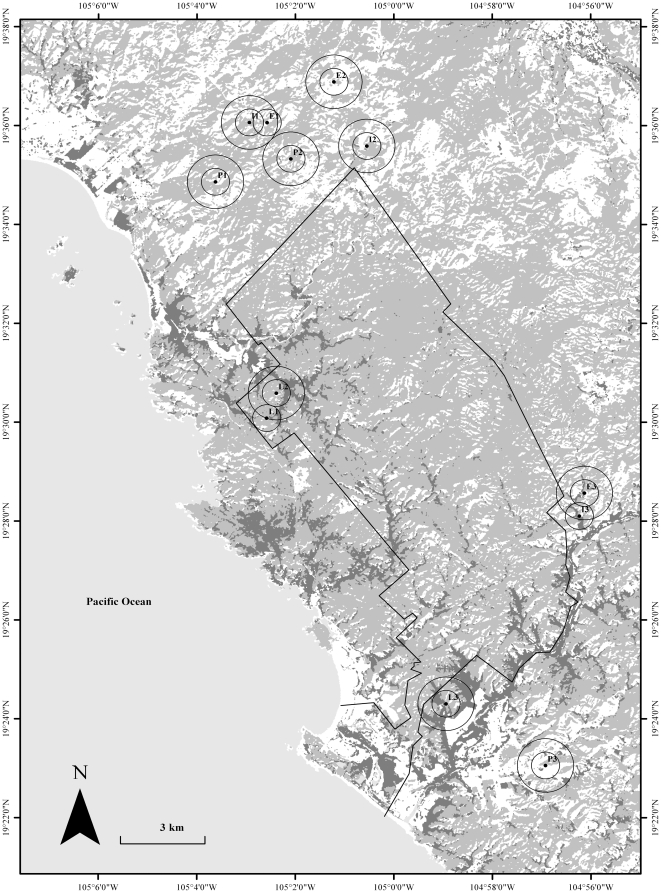
Classified image showing sampling sites and concentric focal scales. Circles around sampling sites represent the focal scales of 500 and 1000 m radii. Successional stages: pasture (P), early (E), intermediate (I) and late (L). Dry forest is colored light gray, whereas small areas of riparian forest are colored dark gray. The polygon encloses the area of the Chamela-Cuixmala Biosphere Reserve.

The study was conducted in and surrounding the Chamela-Cuixmala Biosphere Reserve (CCBR, Fig. 1), located in the central western coast of Mexico in the state of Jalisco (19°22′–19°35′N, 104°56′–105°03′W). The CCBR has an extension of 13,200 ha and is covered by a well preserved tropical dry forest and small areas of riparian forest, among other vegetation types [60]. Its precipitation regime follows a markedly seasonal pattern as most of the rainfall occurs during June–October. Average annual precipitation is 763±258 (SD) mm and average annual temperature is 24.6°C (Chamela Biological Station website: www.ibiologia.unam.mx/ebchamela/, accessed 2008 Feb 1; [27]).

We selected twelve sampling sites (Fig. 1) representing a dry forest successional gradient (chronosequence) of four successional stages differing in their age of abandonment: 3 pastures (0 years), 3 early stage sites (3–5 years), 3 intermediate stage sites (8–12 years) and 3 late stage sites located in the CCBR (at least 50 years old). These sites were selected based on: (1) their slope (ranging from 15° to 25°), allowing the nets to be erected; (2) their aspect, avoiding north facing slopes because of the higher heterogeneity of plant communities occurring on these slopes [61]; (3) their accessibility through trails; (4) their distribution around and distance from the CCBR (pastures, early and intermediate sites were distributed around the reserve in order to generate a research design reasonably balanced; their distance from such reserve was equal or higher than 1000 m). All secondary sites were constituted by plots of 120 * 90 m embedded within the same vegetation type. The land use history of secondary sites is quite similar. First, forest was removed through slash and burn. Second, lands were subsequently used for maize and bean production. Finally, lands were converted into cattle pastures, being burned approximately every two years before rainfall. For more detailed information about the land use history of sampling sites, including pictures, see [27].

### Bat sampling

Bats were captured following a standardized sampling unit consisting of a set of five mist nets covering an area of 109 m^2^ each sampling night. Mist nets were located within and surrounding each plot, crossing natural corridors representing flyways for bats. In pastures, mist nets were located in temporary creeks or in trails delimited by shrubs and treelets. Distance between nets was not shorter than 30 m. Sampling was performed for 5 hours after sunset, a period of time that coincides with the foraging peak for most phyllostomid bats [62], avoiding windy, rainy and full moon nights to reduce variation in capture success. We also avoided biases due to trap-shy behavior by sampling each site for only one night during a census period.

Mist netting remains the single most effective method to sample phyllostomid bat assemblages and to evaluate their response to habitat modification, being widely used in pastures, agricultural fields, secondary vegetation and mature forests [7], [14], [41], [44], [45], [63], [64]. Acoustic sampling was not used as a complementary sampling technique because: (1) phyllostomid bats produce low-intensity calls that can not be clearly detected, and (2) their calls show low inter-specific variation, which precludes species identification [65], [66]. Biases associated with the use of mist nets have been addressed [67], [68], [69].

From June 2004 to August 2006, most sites were sampled every 46±15 (SD) days. In pastures we reduced the number of sampling nights due to the paucity of captures. During each census period we randomized the order in which sites were sampled. Nets were checked approximately every 30 min and captured bats were stored temporarily in cloth bags. Bats were identified to the species level based on the dichotomous keys of Medellín et al. [70] and Timm and Laval [71]. Excluding juveniles and non-healthy individuals, all bats were marked on their forearm using numbered aluminum bands. Individuals were released where they were originally captured. We also collected and identified the seeds found in bat feces inside the cloth bags. In addition, the chiropterophilic and chiropterochoric plants occurring in the region were identified based on literature (see Table S3). We used this information as ancillary data for the discussion of our results, considering which resources are being used by nectarivores and frugivores and how they are distributed in the vegetation matrix. Bat nomenclature follows Simmons [72] and bat ensemble assignation follows Timm and Laval [71].

### Habitat attributes at the local scale

In a quadrate of 50*20 m within each plot, we measured the following vegetation attributes considering all the woody plants with a diameter at breast height equal or greater than 2.5 cm: 1. Number of individuals (IN), 2. Species number (SP), 3. Basal area (BA), and 4. Average leaf area index per plot (LAI), which is the projected green leaf area per unit of a horizontal plane [73]. This index is considered a useful indicator of the biophysical characteristics of vegetation and allows for discrimination of successional stages as its value increases toward sites with higher vegetation complexity (i.e. higher number of strata, woody species, and basal area, [74]). LAI was measured during the rainy season by using an LAI-2000 Plant Canopy Analyzer (LI-COR, USA). Detailed information on how measurements were recorded can be found in Nassar et al. [75].

To obtain a continuous synthetic variable summarizing sampling site variation in vegetation parameters, we performed a principal component analysis (PCA) of the variables described above. All variables were positively correlated with axis 1 of the PCA and their eigenvector values were: IN (0.48), SP (0.52), BA (0.52) and LAI (0.48). Based on this, we assumed that axis 1 represented a successional gradient where the sites with higher vegetation structural complexity (late and intermediate stages) presented higher scores. Axes 1 and 2 explained 83% and 11% of the variation respectively (Fig. S1). Consequently, axis 1 scores were considered as a new variable reflecting the vegetation structural complexity of each site. This new variable was used as an explanatory variable for evaluating bat responses to local scale variation in the habitat.

### Habitat attributes at the landscape scale

The estimation of the landscape metrics used as explanatory variables were performed on a classified image comprised of 4 ASTER, cloud free, satellite images acquired for the Pacific coast of Mexico on December 28, 2005. This date represents an intermediate moment along the bat sampling period and corresponds to the dry season, when the highest differentiation between pastures, dry and riparian forest occurs [76], [77]. For image classification, we analyzed the first three bands of the ASTER sensor as well as two other bands produced by the calculation of two spectral indices: the normalized difference vegetation index (NDVI) and the single ratio (SR), all with a nominal spatial resolution of 15 m. Finally, the image was classified into 9 land-cover classes: (1) dry forest initial successional stages (including pastures and the early successional stage), (2) dry forest advanced successional stages (DF, including intermediate and late successional stages,), (3) riparian forest (RF), including both gallery forest located along large rivers and gallery forest located along temporary creeks, (4) mangroves, (5) oak forest, (6) seasonal growing field (i.e. corn, tomato, hot pepper, watermelon), (7) long term growing field (i.e. mango, papaya, coconut, citrus), (8) bare soil (including dirt and paved roads), and (9) water. The final accuracy of the classified image was 0.86 and 0.84 according to the overall accuracy and Tau coefficient statistics, respectively. The image was processed with ERDAS Imagine v.9.2 (Leica Geosystems, Georgia, USA). More detailed information about image processing can be found in the supporting information (Methods S1).

Landscape metrics were measured at two different focal scales within two nested concentric circles of 500 and 1000 m radius and centered on the centroid of the mist net distributions for each sampling site (Fig. 1). These focal scales allowed us: (1) to encompass the expected home range of small and medium size phyllostomids inhabiting the region (i.e. *Glossophaga soricina*: 500 m radius [78]; *Sturnira lilium*: 636 m radius [79]); (2) to minimize spatial overlap among neighboring circles (sites E1, I3 and L1 were not considered in the 1000 m radius analysis as they were partially overlapped), and (3) to compare our results with other studies [32], [46], [56]. A high degree of overlapping among neighboring circles was avoided in order to minimize the likelihood of making a Type I error (reject a true null hypothesis) due to the effect of pseudoreplication [80], [81]. Pseudoreplication likely would occur if the values of the predictor variables from nearly the same concentric circles were considered as multiple and independent observations in the dataset [82].

Five landscape metrics were selected as explanatory variables based on metrics found in other studies to be associated with the occurrence or abundance of phyllostomids [32], [46], [56]: (1) percentage of dry forest cover (estimated for the DF class, defined above), (2) percentage of riparian forest cover (estimated for the RF class), (3) mean area of dry forest patches (estimated for the DF class), (4) mean area of riparian forest patches (estimated for the RF class) and (5) diversity of patch types. Percentage of forest cover was defined as the sum of the areas (m^2^) of all patches of a given type (DF or RF) divided by the total area of the plot (circle) and multiplied by 100. Mean patch area was defined as the sum of the areas (m^2^) of all patches of a given type (DF or RF) divided by the number of patches of such type. Diversity of patch types was defined as the probability that any two cells selected at random would represent different patch types (Simpson's diversity index). All coverage classes considered in the image classification were used in the estimation of patch type diversity. Calculation of landscape metrics was performed using Fragstat v.3.3 [83].

### Statistical Analyses

The completeness of bat surveys in all sampling sites was assessed by calculating the percentage of the total estimated species richness that effectively was covered by samples. Total species richness was estimated by computing the mean of the first and second order Jackknife indices [84]. These indexes deal properly with small sample sizes (<100 individuals per site), producing a low biased estimation of species richness [85]. Ninety percent of completeness was considered an appropriate level of sampling efficiency [86].

We tested our data for spatial autocorrelation using a Mantel test (999 permutations) based on Spearman rank correlation coefficients for ecological and geographic distance matrices. We assessed significance of spatial structure at an alpha = 0.05 level. The Bray-Curtis coefficient [84] was used for the construction of the ecological distance matrix, and the Euclidian distance between sites for the construction of the geographic distance matrix.

The response of phyllostomids to habitat variation at local and landscape scales was evaluated at the population, ensemble and assemblage-level. In all cases we used capture rate (individuals/night) as an indicator of local abundance. Only the seven most common species (n≥25) were analyzed at the population-level. We also considered as a response variable, at ensemble and assemblage-level, the number of captured species, which was rarified at 8 sampling nights (EstimateS software v 8: http://viceroy.eeb.uconn.edu/estimates). Rarefaction allows the comparison of indices by eliminating biases related to differences in sampling effort [87].

Dissimilarities between assemblages in terms of species identity and abundance were quantified using the Bray-Curtis coefficient, which is one of the preferred dissimilarity measures as it adequately reflects the intuitive ordering of sites [88]. However, the value of this coefficient is more influenced by the species with the largest difference in abundance and consequently, in a dataset dominated by a few species, this coefficient will mainly reflect differences for those species [88]. To avoid this bias, the matrix of sampling sites by species abundance was standardized using the square-root. All phyllostomids were considered in the analysis.

The distance matrix produced was used as an input matrix for a non-metric multidimensional scaling ordination (NMDS), which maps the observed assemblage's dissimilarities in species composition. This iterative method of ordination has the advantage of properly handling nonlinear species response of any shape [89] and has a good performance even when beta diversity is high [90]. The scores of the resulting axes were employed as a response variable to evaluate the relationship between the variation in assemblage species composition and the variation in habitat attributes.

We evaluated the relationship between the phyllostomid response and all the explanatory variables, at the 500 and 1000 m radius scales, using generalized linear models (GLMs). The NMDS scores and the rarified number of species at the ensemble and assemblage-level were modeled using a Gaussian error distribution with the identity link function, as this is the error distribution that best describes the structure of the data. The abundance data at the population, ensemble and assemblage-level were modeled using a Poisson error distribution with the log link function.

As in previous studies, we found multicollinearity problems because the metrics of landscape structure are correlated with the percentage of cover of the corresponding land-cover class [32], [55]. In order to avoid this problem, in all subsequent analyses we substituted the estimated values of mean patch area by the residual values of the regression between this variable and the percentage of cover [32].

The explanatory variables most likely to causally influence the response variables at the two focal scales were identified by performing a hierarchical partitioning analysis (HPA) [91]. In this analysis, all possible GLMs combining the explanatory variables are jointly considered and the increase in model fit generated by certain variables (measured by the log-likelihood) is estimated by averaging the variables influence over all models in which such variables appear [91], [92]. At the end, we obtained a measure of the independent effect of each explanatory variable over the response variable. This procedure alleviates problems of multicollinearity between the explanatory variables, which can not be properly handled by statistical sequential methods (i.e. stepwise selection). Sequential methods would tend to select spurious models due to a high type-I error [92]. Significance of relationships (α = 0.05) between explanatory and response variables was evaluated by the randomization test suggested by Mac Nally [93]. We compared the HPA outcomes using the small-corrected form of AICc for model selection [94] in order to validate their robustness [92]. The set of models considered included the null model and six other models considering each explanatory variable independently. More detailed information can be found in the supporting information (Table S2).

Finally, based on the results of HPA and model selection based on AIC, we classified the relationships between every explanatory variable and the corresponding response variables as: (1) robust: when a significant relationship was found (HPA) and when the explanatory variable was selected as part of the most plausible models (AIC), (2) those denoting a tendency: when the explanatory variables were associated with the greatest portion of the variation in the response variable, although no significant relationship was observed (HPA), and they were selected as part of the most plausible models (AIC), and (3) no relationship: when the explanatory variables were not associated with the greatest portion of the variation in the response variable (HPA), and/or they were not selected as part of the most plausible models (AIC).

All the statistical analyses were performed in R [95] using vegan [96], MASS [97] and hier.part packages [98].

## Results

One hundred and forty-two sampling nights resulted in the capture of 606 phyllostomid individuals representing 15 species, 11 genera, 5 subfamilies and 4 broad guilds (Table 1). The most abundant species were *Artibeus jamaicensis*, *G. soricina* and *Desmodus rotundus* whereas the broad guilds best represented in terms of species richness were frugivores and nectarivores. The number of captures per species and the sampling effort per site can be found in Avila-Cabadilla et al. [27]. Individuals previously identified as *A. intermedius* (Table 2 on [27]), were re-classified as *A. lituratus* following Simmons [72] (Table 1).

**Table 2:** Relationships between population, ensemble and assemblage-level parameters and the habitat attributes.

		Type of relationships between variables	
Parameter	Scale	Robust	Tendency
**Population level**			
Nectarivore			
*G. soricina*	500		DF_area_(-); Div
*G. commissarisi*	500		DF_area_(-)
	1000		DF_area_(-)
*L. yerbabuenae*	1000		DF_area_(-)
Frugivore			
*A. jamaicensis*	500	V_struct_	RF_%_
	1000		DF_area_(-)
*A. phaeotis*	500		V_struct_; RF_%_
	1000		RF_%_
*A. lituratus*	500	RF_%_	
	1000		DF_area_; RF_%_
Sanguivore			
*D. rotundus*	500	RF_%_	
	1000		RF_area_
**Ensemble-level**			
Nectarivore			
AbN	500		DF_area_(-)
	1000		DF_area_(-)
Frugivore			
S_8_F	500	V_struct_	
	1000		V_struct_
AbF	500	RF_%_	
	1000	RF_%_	
**Assemblage-level**			
SC_2_	500	V_struct_(-); RF_%_(-)	
	1000		RF_%_(-)
AbP	500	RF_%_	
	1000	RF_area_	

Parameter at population-level: species abundance. Parameters at ensemble-level: rarified number of nectarivorous (S_8_N) and frugivorous species (S_8_F), abundance of nectarivores (AbN) and frugivores (AbF). Parameters at assemblage-level: scores of the first (SC_1_) and second ordination axis (SC_2_) reflecting assemblage dissimilarities in species composition and structure, rarified number of phyllostomid species (S_8_P) and abundance of phyllostomids (AbP). Habitat attributes: vegetation structural complexity (V_struct_), mean area of dry (DF_area_) and riparian forest patches (RF_area_), percentage of riparian forest cover (RF_%_) and diversity of patch types (Div). Negative relationships are shown in parentheses.

Sampling effort was considered sufficient to characterize phyllostomid assemblages occurring in each sampling site. Completeness reached 90% in all cases, ranging from 90 (P1, E2) to 96% (I2, I3). We found no evidence of spatial structure in our dataset. Ecological and Euclidian distance matrices were not significantly correlated (r_s_ = −0.05, p = 0.59).

### Phyllostomid response at the population-level

Summarizing the results of both statistical analyses (see Table S1 for HPA and Table S2 for AIC), we found that variation in abundance of the three nectarivorous species was not robustly associated with the variation of any explanatory variable at the two focal scales (Table 2), although it tended to be negatively associated with variation in the mean area of DF patches (at the two scales). We also identified a tendency for a positive association between *G. soricina* abundance and the diversity of land cover types (Table 2). On the other hand, while *G. soricina* only responded to changes in habitat attributes at the 500 m focal scale, *Leptonycteris yerbabuenae* only responded to such changes at the 1000 m focal scale.

Most of the variation in abundance of frugivorous species showed a robust, positive, association with variations in percentage of RF (all species) and complexity of vegetation structure (*A. jamaicensis* and *A. phaeotis*). We also detected, at the 1000 m focal scale, that the mean area of DF patches tended to be associated with the abundance of both frugivores, *A. jamaicensis* (negative association) and *Artibeus lituratus* (positive association) (Table 2).

The abundance of the sanguivorous *D. rotundus*, showed a robust, positive association with variations in the percentage of RF (at the 500 m focal scale) and tended to be positively associated to the mean area of RF patches (at the 1000 m focal scale) (Table 2).

### Phyllostomid response at the ensemble-level

Similarly to what we observed at the population-level, the variation in the abundance of nectarivores tended to be negatively associated, at the two scales, with variations in the mean area of DF patches. No explanatory variable was associated with the variation in the number of nectarivorous species, whereas the variation in the number of frugivorous species was positively associated with variations in the complexity of vegetation structure. The abundance of frugivores was robustly and positively associated with the percentage of RF at the two scales.

### Phyllostomid response at the assemblage-level

**Figure 2:**
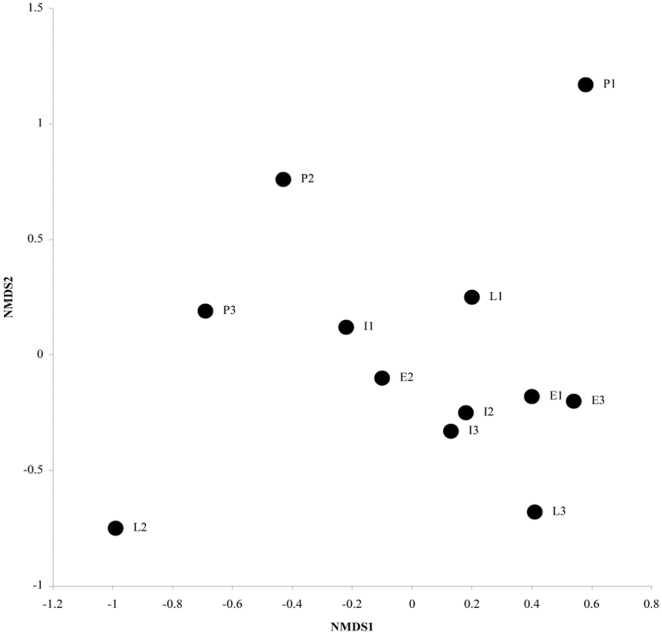
Ordination of sampling sites based on the species composition and structure of phyllostomid assemblages. Successional stages: pasture (P), early (E), intermediate (I) and late (L). NMDS 1 and 2: axis 1 and 2 of the non-metric multidimensional scaling.

Only two axes were considered in the NMDS ordination of phyllostomid assemblages (Fig. 2, stress = 8.20), because additional dimensions did not substantially diminish the stress value. A successional gradient is represented along axis 2, where phyllostomid assemblages occurring in pastures tended to present higher scores and those occurring in late stages tended to present lower scores. Axis 1 did not show a clear gradient.

The assemblage dissimilarities represented by NMDS axis 2 (Fig. 2) were significantly associated with the gradient of vegetation structural complexity found along PCA axis 1 (Fig. S1) at the 500 m scale (Table 2). NMDS axis 2 was also negatively associated with the percentage of RF at the two scales (Table 2). None of the considered explanatory variables were associated with NMDS axis 1.

The variation in phyllostomid abundance was positively associated with the percentage of RF (at the 500 m scale) and with the mean area of RF patches (at the 1000 m scale). No explanatory variables were associated with the variation in the number of phyllostomid species.

## Discussion

### Phyllostomid response at the population and ensemble-level

As expected, we found a guild-specific response of bats to changes in local and landscape habitat attributes. First, the abundance of nectarivores, at both the population and ensemble-level, tended to decrease with the increase in the mean area of dry forest patches, while the number of species was not associated with any of the explanatory variables. On the other hand, frugivore abundance was tightly and positively associated with the percentage of riparian forest while the number of species and some species abundances (*A. jamaicensis* and *A. phaeotis*) were associated with variation in the vegetation structural complexity. Finally, the abundance of the unique sanguivore present in the region (*D. rotundus*) increased with the increase in the amount of riparian forest. Guild-specific bat responses previously have been found by Klingbeil and Willig [56] and Henry et al. [52] in different Amazonian rainforests. In both studies the authors consistently found that the frugivore abundance responded more to changes in landscape composition (i.e. percentage of forest cover), while gleaning animalivorous bats responded more to changes in landscape configuration (i.e. edge density). The guild-specific response of bats to variation in habitat attributes is likely due to the contrasting ecological requirements of bats from different ensembles.

The lack of relationship between the number of nectarivorous species and the considered habitat attributes probably occurs because three of the five nectarivores reported in this study are ubiquitous. These species (*G. soricina*, *G. commissarisi* and *L. yerbabuenae*) were present in most of the sampling sites and successional stages with the exception of pastures, where only 3 individuals of *G. soricina* were captured [27]. On the other hand, the two species most susceptible to variation in habitat attributes (*C. godmani* and *M. harrisoni*), which were strictly associated with the less disturbed areas, were scarcely represented (one individual) in two of the three sites of the late successional stage [27].

The negative association between nectarivore abundance and the mean area of dry forest patches, probably reflects the behavior of the three most abundant nectarivores (*G. soricina*, *G. commissarisi* and *L. yerbabuenae*) which are not so dependent on large dry forest patches. Naturally, these species are well adapted to inhabit regions where vegetation has a simple structure, like deserts, arid grasslands, scrublands, and lowland dry forest [99], [100], [101], [102], [103], being able to exploit the trophic resources available in these areas (i.e. plants of the family Cactaceae and Asparageceae, [25], [104]). In the study region, these three nectarivorous species occur at a higher abundance in the early successional stage, characterized by the presence of non-native grasses, shrubs and treelets, and where some potential chiropterophilic species (i.e. *Acacia farnesiana*, *Cordia alliodora*) are highly abundant [27], [105], [106], [107]. Other studies have reported that the abundance of nectarivores with a more generalist feeding and habitat preferences (i.e. *G. soricina*, *G. commissarisi*, *Lonchophylla robusta*, *Monophyllus redmani* and *Phyllonycteris poeyi*), appears unaffected or increases in disturbed and secondary vegetation [14], [30], [31], [37], [41], [44], [46], [108], [109], [110], [111].

In contrast to the nectarivores' response, the number of frugivorous species, as well as the abundance of some of these species, increased toward the more advanced successional stages, which present the highest vegetation structural complexity (Fig. S1, Table 2). Indeed, in pastures, the sites with the simplest vegetation structure, only three species of frugivores occurred (*A. jamaicensis*, *A. watsoni*, and *A. lituratus*) while in the late successional stage, 8 species occurred, three of them (*Carollia sp.*, *Centurio senex* and *Chiroderma salvini*) exclusively in this stage [27]. These responses can be explained by an increase in the diversity and amount of resources (i.e. food and shelter) with the increase in the complexity of vegetation structure. The loss of trees due to clearing for grazing and agriculture can negatively influence frugivore abundance in the Neotropics by affecting their ability to locate suitable roosting sites, as they preferentially roost in hollow trees found in mature forest or in advanced successional stages [11], [112], [113]. In the tropical dry forest, the diversity and amount of trophic resources for frugivores decrease toward the early successional stage, dominated by anemochorous and autochorous plants which do not constitute food resources for these species [114] (P. Balvanera et al. unpublished data). This contrasts with the patterns found in tropical humid and rain forest, where chiropterochorous species such as *Cecropia* spp., *Piper* spp., *Solanum* spp., and *Vismia* spp., are dominant in the early stages of succession [11], [37].

**Figure 3:**
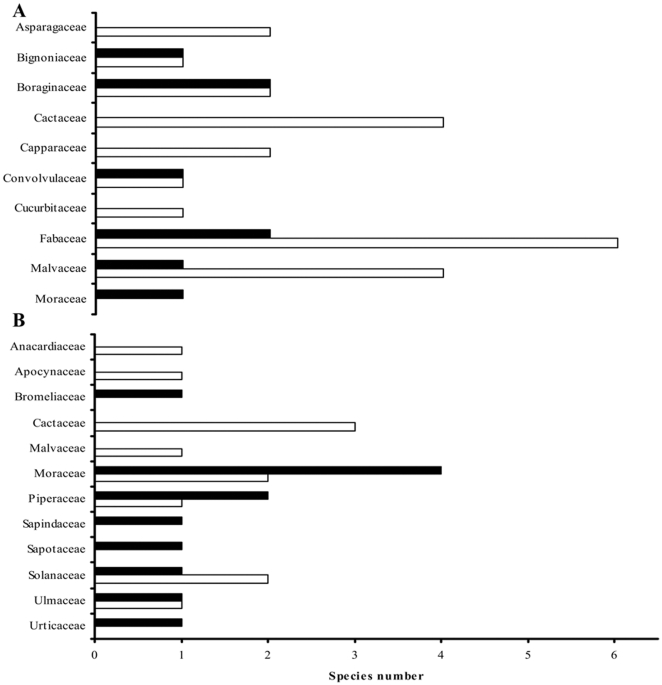
Number of chiropterophylic and chiropterochoric species per plant family occurring in dry and riparian forest. A: chiropterophylic species, B: chiropterochoric species. Dry and riparian forests arerepresented by white and black bars respectively. The entire species' checklist of the Chamela-Cuixmala region, as well as detailed information on how it was generated, appear in Table S3.

Nectarivores and frugivores showed a marked difference in their response to variations in landscape attributes. Variation in nectarivore abundance tended to be associated with variation in landscape attributes concerning dry forest vegetation while variation in frugivore abundance was tightly associated to variation in landscape attributes concerning riparian forest vegetation. This probably occurs because dry and riparian forests differ in terms of the availability of resources for both guilds. Although both forests host an equal number of chiropterochorous plant families and species (8 families, 12 species), the plants preferentially used by most of the frugivorous species (i.e. Moraceae and Piperaceae are heavily consumed by *Artibeus* and *Carollia*, respectively) are almost exclusively found in riparian forest (Fig. 3, Table S3, [26]). This explains why the abundance of the three frugivores analyzed, as well as the overall abundance of frugivorous bats, was higher in sites with a greater percentage of riparian forest. An analogous result has been reported for tropical rain forests, where the variation in the abundance of a chiropterocoric resource, *Piper* spp., was significantly and positively associated with variations in the abundance of shrub frugivorous bats [51]. In contrast, most chiropterophilic species (23 species from 9 families) as well as the chiropterochorous Cactaceae species, important food resources for nectarivorous bats, occur in the dry forest, within both the early and more advanced successional stages (Fig. 3, Table S3, [26], [115]). Only 8 chiropterophilic species, representing 6 plant families, occur in the riparian forest. Consequently, dry forest vegetation hosts more plant species that constitute resources for nectarivores than riparian forest vegetation (Fig. 3).

The higher abundance of the sanguivorous *D. rotundus* toward sites with a greater amount of riparian vegetation, can occur because: 1) the riparian forest offers higher roost availability for this species [116], [117] and/or it uses this habitat as stepping stones when searching for food in the vegetation matrix, as suggested by Estrada and Coates-Estrada [40] for explaining how this species uses forested areas when searching for food in pasture-dominated landscapes; 2) there is a higher availability of sanguivores' native food sources (mammals) in the riparian vegetation. Riparian forests contain important resources for the maintenance and connectivity of the home ranges of medium and large-sized mammals [118], [119], [120]. Moreover, some mammal species concentrate their activities in riparian forest, especially during the dry season when most resources are limited in the region [77]; 3) the riparian forest also hosts a higher amount of non-native source of food (cattle), as it occurs on alluvial terraces along the channels of ephemeral and permanent streams. Farmers concentrate the cattle in these areas as they constitute the most important sources of water in the region; the cattle concentration in these areas reaches its highest point during the dry season (Pers. Obs).

### Phyllostomid response at the assemblage-level

As expected, a portion of the variation among phyllostomid bat assemblages, in terms of species composition and structure (NMDS axis 2, Fig. 2), was tightly associated with the variation in vegetation structural complexity and vegetation cover (riparian forest). Moreover, phyllostomid abundance was significantly associated with the percentage of riparian forest (at 500 m scale) and with the mean area of riparian forest patches (at 1000 m scale). These results integrate the results obtained at the species and ensemble-level.

The association between assemblage characteristics and vegetation structural complexity has some potential explanations. First, a group of species is found exclusively in sites with a determined vegetation structure. This is the case for rare species (represented by one individual) and some species with an intermediate level of abundance (*Micronycteris microtis*, *Musonycteris harrisoni*, *Choeroniscus godmani*, *Carollia* sp., *Centurio senex*, *Chiroderma salvini*), which are strictly associated with the late successional stage [27]. In fact, *M. microtis*, *M. harrisoni*, *C. godmani and C. senex* already have been identified as forest dwelling species [14], [41], [49], [50], [106]. Second, variation of some species' abundance is tightly associated with variation in vegetation structure. This is the case for some of the most abundant frugivorous species. As discussed above, these responses could follow variations in availability of roosting sites and/or food as a consequence of structural changes.

The association between the amount of riparian forest and phyllostomid assemblage composition and overall abundance is mainly a consequence of frugivore response, as they constitute the best represented ensemble (in terms of species richness and number of captured individuals) and their abundance tends to increase towards sites with a greater amount of riparian forest.

### Implications for the study of phyllostomid bats

In accordance with previous studies [32], [46], [52], [56], our results showed that bat responses to habitat modification are scale-dependent. Variation in some explanatory variables was associated with variation in some response variables at just one of the two analyzed scales (Table 2). Such dependence can be due to the species-specific degree of mobility, habitat requirements and life-history characteristics [56]. These results indicate that a single scale approach may be inadequate for understanding bat responses to habitat modifications and that a multi-spatial scale approach must be employed. Hence, the spatial scale must be taken into account when determining habitat attributes whose variation defines bat responses in a transformed landscape. The marked difference between the ensemble's response to landscape attributes also indicates the necessity of considering different land-cover classes when characterizing landscape attributes. With the exception of one study [121], a multi-land-cover class approach has not been considered in most of the related bat studies in the Neotropics.

The guild-specific association between bat abundance variation and variation in landscape features could be used for modeling the variation in the functional diversity of phyllostomid bat assemblages across anthropogenic landscapes [52]. This would represent an invaluable tool allowing us to identify the most important areas for preserving the ecological services provided by these bats (i.e. pollination, seed dispersal). The abundance-based modeling of variations in functional diversity can also be used as a surrogate for modeling variation in species richness, a widely used indicator for conservation planning. This approach would be highly useful especially when species diversity parameters cannot be accurately estimated in all sampled sites due to small sample sizes [52].

Our results also point out the importance of comparing outcomes from different statistical analyses in order to identify the most relevant associations among bat responses and variation in habitat attributes. The two statistical analyses we employed (HPA and AIC), for example, have recently been used for identifying habitat attributes whose variation may explain bat response to habitat modifications [46], [56], but the results we obtained from each statistical analysis were different in certain cases.

### Implications for phyllostomids bats conservation

As in previous studies, our results reflect phyllostomid bats sensitivity to habitat loss or modification at both local and landscape levels [8], [32], [37], [39], [41], [44], [56]. Long-term conservation of phyllostomids in anthropogenic landscapes only can be achieved by realizing that management of the habitat at the landscape level (regarding its composition and configuration) is as important as the conservation of particular forest fragments. In this sense, to guarantee the conservation of phyllostomids in tropical dry forest landscapes the conservation and restoration of riparian forest should be prioritized. This habitat and its surrounding areas have been extensively affected by agricultural and cattle production, as they represent the main source of water and fertile soils in tropical dry forest regions [77], [122]. Bat abundance at the species, ensemble and assemblage-levels is negatively affected by reductions in the amount of riparian forest as this habitat represents an important source of trophic resources for a great number of phyllostomids, especially for the frugivores (Fig. 3). The amount of riparian forest can be even more critical for bat assemblages during the dry season, when availability of food is limited in dry forest and most resources are concentrated in riparian forest [77]. In addition, riparian forest can play an important role in the maintenance and connectivity of bats' home ranges functioning as a vegetation corridor across the agricultural landscape, providing physical connectivity among isolated forests or acting as stepping stones [45], [57].

Land-use policies must also focus on the maintenance of large areas of mature forest, as several species appear to be greatly associated with sites with a high vegetation structural complexity. The maintenance of mature forests appears to be critical for rare species which, due to their specialized foraging requirements and restricted mobility, are unable to move and use the resources available in other habitats [27], [40]. Finally, we suggest that policies must consider the inclusion of secondary vegetation in conservation areas/programs [123], as suggested by the “Red de Areas Ejidales Protegidas" proposed by Sanchez-Azofeifa et al. [77]. Several phyllostomids occur, and presumably exploit the resources available in such areas. In fact, some species of nectarivores show a higher abundance in secondary vegetation compared to mature forest. Consequently, secondary vegetation can be considered as an important habitat for preserving bat diversity.

## Supporting Information

Figure S1 **Ordination of sampling sites based on the structural attributes of vegetation.**
(DOC)

Table S1 **Percentage of variation in population, ensemble and assemblage-level parameters, associated with the variation of the habitat attributes.**
(DOC)

Table S2 **Most plausible models (95%) explaining the variation in population, ensemble and assemblage-level parameters.**
(DOC)

Table S3 **Chiropterophylic and chiropterochoric species occurring in the Chamela-Cuixmala region.**
(DOC)

Methods S1 **Description of the image classification process.**
(DOC)
