# Supplementary material for: Local and Landscape Factors Determining Occurrence of Phyllostomid Bats in Tropical Secondary Forests
Source: PLoS One. 2012 Apr 18;7(4):e35228. doi: 10.1371/journal.pone.0035228 (PMC3329449; doi:10.1371/journal.pone.0035228)
Supplement: Figure S1 — Ordination of sampling sites based on the structural attributes of vegetation. (DOC) [file pone.0035228.s001.doc]

Figure S1. Ordination of sampling sites based on the structural attributes of vegetation.

Successional stages: pastures (P), early (E), intermediate (I) and late (L). PCA 1 and 2: axis 1 and 2 of the principal component analysis.

**PCA2**

**PCA1**

P3

P1

P2

E2

E3

E1

I3

I2

L1

L2

I1

L3
