# Supplementary material for: Local and Landscape Factors Determining Occurrence of Phyllostomid Bats in Tropical Secondary Forests
Source: PLoS One. 2012 Apr 18;7(4):e35228. doi: 10.1371/journal.pone.0035228 (PMC3329449; doi:10.1371/journal.pone.0035228)
Supplement: Table S1 — Percentage of variation in population, ensemble and assemblage-level parameters, associated with the variation of the habitat attributes. (DOC) [file pone.0035228.s002.doc]

Table S1. Percentage of variation in population, ensemble and assemblage-level parameters associated with the variation of the habitat attributes.

|  |  |  |  |  | **Habitat attribute** | | | | |
| --- | --- | --- | --- | --- | --- | --- | --- | --- | --- |
| **Parameter** | **Scale** | ***n*** | $R_{dev}^{2}$ | **V_struct_** | **DF%** | **DF_area_** | **RF%** | **RF_area_** | **Div** |
| **Population-level** |  |  |  |  |  |  |  |  |  |
| Nectarivores |  |  |  |  |  |  |  |  |  |
| *G. soricina* | 500 | 12 | 0.17 | 7.34 (-) | 8.79 | 40.65 (-) | 7.94 | 5.19 | 30.09 |
|  | 1000 | 9 | 0.70 | 4.75 | 6.97 | 35.09 (-) | 10.11 | 30.23 | 12.85 |
| *G. commissarisi* | 500 | 12 | 0.50 | 15.19 (-) | 2.80 | 49.79 (-) | 11.18 | 6.45 | 14.59 |
|  | 1000 | 9 | 1.00 | 16.23 | 12.81 (-) | 48.40 (-) | 6.28 (-) | 7.97 | 8.30 |
| *L. yerbabuenae* | 500 | 12 | 0.10 | 5.73 (-) | 34.23 | 9.82 (-) | 4.55 (-) | 1.17 (-) | 44.50 |
|  | 1000 | 9 | 1.00 | 2.03 | 1.56 (-) | 11.23 (-) | 2.37 | 4.36 | 1.43 (-) |
| Frugivores |  |  |  |  |  |  |  |  |  |
| *A. jamaicensis* | 500 | 12 | 0.86 | **36.70** | 14.58 (-) | 6.74 | 28.08 | 2.43 (-) | 11.47 (-) |
|  | 1000 | 9 | 1.00 | 20.24 | 13.64 (-) | 20.58 (-) | 9.42 | 17.86 | 18.26 (-) |
| *A. phaeotis* | 500 | 12 | 0.79 | 29.67 | 11.98 | 5.03 | 25.76 | 2.72 | 24.84 |
|  | 1000 | 9 | 1.00 | 17.37 | 8.85 (-) | 18.94 (-) | 23.35 | 16.17 (-) | 15.32 (-) |
| *A. lituratus* | 500 | 12 | 0.84 | 32.89 | 7.08 (-) | 3.43 | **36.96** | 15.61 | 4.03 (-) |
|  | 1000 | 9 | 1.00 | 17.61 | 4.89 (-) | 28.81 | 24.00 | 15.80 (-) | 8.90 (-) |
| Sangivorous |  |  |  |  |  |  |  |  |  |
| *D. rotundus* | 500 | 12 | 0.88 | **41.28** | 3.85 (-) | 3.31 (-) | **44.73** | 3.31 (-) | 3.52 (-) |
|  | 1000 | 9 | 1.00 | 18.93 | 7.62 (-) | 21.42 (-) | 13.73 | 23.87 | 14.43 (-) |
| **Ensemble-level** |  |  |  |  |  |  |  |  |  |
| Nectarivores |  |  |  |  |  |  |  |  |  |
| S_8_N | 500 | 11 | 0.30 | 14.74 (-) | 2.72 | 43.14 (-) | 5.33 | 32.83 (-) | 1.24 |
|  | 1000 | 8 | 0.94 | 14.43 | 12.74 (-) | 20.18 (-) | 12.81 | 28.01 | 11.83 (-) |
| AbN | 500 | 12 | 0.18 | 5.92 (-) | 9.47 | 42.63 (-) | 6.48 | 5.09 | 30.40 |
|  | 1000 | 9 | 0.74 | 6.54 | 7.18 | 46.85 (-) | 9.05 | 18.41 | 11.97 |
| Frugivores |  |  |  |  |  |  |  |  |  |
| S_8_F | 500 | 11 | 0.90 | **26.06** | 12.10 (-) | 13.30 (-) | **24.43** | 17.43 | 6.69 (-) |
|  | 1000 | 8 | 0.95 | 31.68 | 12.33 (-) | 18.66 (-) | 12.58 | 15.08 | 9.67 (-) |
| AbF | 500 | 12 | 0.75 | 23.41 | 2.30 (-) | 2.47 (-) | **54.44** | 14.74 | 2.64 (-) |
|  | 1000 | 9 | 1.00 | 20.73 | 6.27 (-) | 18.99 (-) | **39.89** | 7.79 (-) | 6.34 (-) |

Table S1 (continue)

|  |  |  |  |  | **Explanatory variable** | | | | |
| --- | --- | --- | --- | --- | --- | --- | --- | --- | --- |
| **Parameter** | **Scale** | ***n*** | $R_{dev}^{2}$ | **V_struct_** | **DF%** | **DF_area_** | **RF%** | **RF_area_** | **Div** |
| **Assemblage-level** |  |  |  |  |  |  |  |  |  |
| SC_1_ | 500 | 12 | 0.26 | 5.02 (-) | 15.53 | 35.37 (-) | 3.74 (-) | 36.48 (-) | 3.87 |
|  | 1000 | 9 | 0.64 | 2.98 (-) | 9.52 | 18.22 (-) | 7.31 (-) | 50.46 | 11.50 |
| SC_2_ | 500 | 12 | 0.81 | **37.47** (-) | 19.91 | 2.07 (-) | **32.69** (-) | 2.83 (-) | 5.04 |
|  | 1000 | 9 | 0.94 | 28.80 (-) | 14.82 | 8.33 | 28.68 (-) | 7.22 | 12.15 |
| S_8_P | 500 | 11 | 0.60 | 28.65 | 12.21 (-) | 10.51 (-) | 36.18 | 8.57 | 3.84 (-) |
|  | 1000 | 8 | 0.98 | 21.09 | 15.40 (-) | 16.05 (-) | 23.37 | 9.31 | 14.79 (-) |
| AbP | 500 | 12 | 0.72 | 30.66 | 5.99 (-) | 9.00 (-) | 38.48 | 4.30 | 11.57 |
|  | 1000 | 9 | 0.91 | 18.25 | 9.02 (-) | 19.45 (-) | 16.70 | 20.16 | 16.42 (-) |

Parameters at population-level: capture rate (individuals/night) as indicator of local abundance. Parameters at ensemble-level: rarified number of nectarivorous (S_8_N ) and frugivorous species (S_8_F); and capture rate of nectarivores (AbN) and frugivores (AbF). Parameters at assemblage-level: scores of the first (SC_1_) and second (SC_2_) ordination axis reflecting assemblages’ dissimilarities in species composition and structure; rarified number of phyllostomid species (S_8_P); and capture rate of phyllostomid (AbP). Habitat attributes: vegetation structure complexity (V_struct_); mean area of dry (DF_area_) and riparian forest patches (RF_area_); percentage of dry (DF%) and riparian forest cover (RF%); and diversity of patch types (Div). n: number of sampling sites. $R_{dev}^{2}$ is the fraction of the total deviance explained by a model considering all explanatory variables when the Poisson error distribution was used and $R^{2}$ when the normal error distribution was used. Significant relationships according to the randomization test appear in bold. Negative relationships are shown in parentheses.
