# Supplementary material for: Local and Landscape Factors Determining Occurrence of Phyllostomid Bats in Tropical Secondary Forests
Source: PLoS One. 2012 Apr 18;7(4):e35228. doi: 10.1371/journal.pone.0035228 (PMC3329449; doi:10.1371/journal.pone.0035228)
Supplement: Table S2 — Most plausible models (95%) explaining the variation in population, ensemble and assemblage-level parameters. (DOC) [file pone.0035228.s003.doc]

Table S2 Most plausible models (95%) explaining the variation in population, ensemble and assemblage-level parameters.

| **Parameter** | **Scale** | **Model** | ***K*** | ***logLik*** | ***AIC_c_*** | **Δ_i_** | ***w_i_*** |
| --- | --- | --- | --- | --- | --- | --- | --- |
| **Population-level** |  |  |  |  |  |  |  |
| Nectarivore |  |  |  |  |  |  |  |
| *G. soricina* | 500 | DF_area_ | 2 | -54.21 | 113.74 | 0.00 | 0.68 |
|  |  | Div | 2 | -56.16 | 117.66 | 3.91 | 0.10 |
|  | 1000 | RF% | 2 | -40.40 | 86.81 | 0.00 | 0.38 |
|  |  | RF_area_ | 2 | -40.78 | 87.55 | 0.74 | 0.26 |
| *G. commissarisi* | 500 | DF_area_ | 2 | -26.78 | 58.90 | 0.00 | 0.72 |
|  | 1000 | DF_area_ | 2 | -23.08 | 52.15 | 0.00 | 0.65 |
| *L. yerbabuenae* | 1000 | DF_area_ | 2 | -10.78 | 27.57 | 0.00 | 1.00 |
| Frugivore |  |  |  |  |  |  |  |
| *A. jamaicensis* | 500 | RF% | 2 | -76.10 | 157.53 | 0.00 | 0.90 |
|  |  | V_struct_ | 2 | -78.30 | 161.94 | 4.41 | 0.10 |
|  | 1000 | DF_area_ | 2 | -38.58 | 83.16 | 0.00 | 0.99 |
| *A. phaeotis* | 500 | V_struct_ | 2 | -17.11 | 39.56 | 0.00 | 0.59 |
|  |  | RF% | 2 | -17.49 | 40.31 | 0.75 | 0.40 |
|  | 1000 | RF% | 2 | -8.18 | 22.36 | 0.00 | 1.00 |
| *A lituratus* | 500 | RF% | 2 | -21.78 | 48.90 | 0.00 | 0.95 |
|  | 1000 | DF_area_ | 2 | -15.40 | 36.81 | 0.00 | 0.50 |
|  |  | RF% | 2 | -15.45 | 36.91 | 0.10 | 0.48 |
| Sangivore |  |  |  |  |  |  |  |
| *D. rotundus* | 500 | RF% | 2 | -37.55 | 80.43 | 0.00 | 1.00 |
|  | 1000 | RF_area_ | 2 | -18.31 | 42.62 | 0.00 | 0.91 |
| **Ensemble-level** |  |  |  |  |  |  |  |
| Nectarivore |  |  |  |  |  |  |  |
| AbN | 500 | DF_area_ | 2 | -91.95 | 189.24 | 0.00 | 0.95 |
|  | 1000 | DF_area_ | 2 | -74.45 | 154.90 | 0.00 | 0.46 |
|  |  | RF% | 2 | -74.70 | 155.40 | 0.50 | 0.36 |
| Frugivore |  |  |  |  |  |  |  |
| S_8_F | 500 | V_struct_ | 3 | -14.06 | 37.55 | 0.00 | 0.40 |
|  | 1000 | V_struct_ | 3 | -7.14 | 26.29 | 0.00 | 0.80 |
| AbF | 500 | RF% | 2 | -19.29 | 43.91 | 0.00 | 1.00 |
|  | 1000 | RF% | 2 | -13.35 | 32.70 | 0.00 | 0.99 |
| **Assemblage-level** |  |  |  |  |  |  |  |
| SC_2_ | 500 | RF% | 3 | -5.97 | 17.93 | 0.00 | 0.51 |
|  |  | V_struct_ | 3 | -6.53 | 19.06 | 1.13 | 0.29 |
|  | 1000 | RF% | 3 | -4.49 | 19.79 | 0.00 | 0.54 |
| AbP | 500 | RF% | 2 | -133.37 | 272.08 | 0.00 | 1.00 |
|  | 1000 | RF_area_ | 2 | -104.52 | 215.03 | 0.00 | 0.99 |

Parameters at population-level: capture rate (individuals/night) as indicator of local abundance. Parameters at ensemble-level: rarified number of frugivorous species (S_8_F) and capture rate of nectarivores (AbN) and frugivores (AbF). Parameters at assemblage-level: scores of the second ordination axis (SC_2_) reflecting assemblage’s dissimilarities in species composition and structure, and capture rate of phyllostomids (AbP). Habitat attributes: vegetation structural complexity (V_struct_); mean area of dry and riparian forest patches (DF_area_ and RF_area_ respectively), percentage of riparian forest cover (RF%) and diversity of patch types (Div). *K*: number of estimated parameters, *logLik*: log-likelihood, *AIC_c_*: sample-size adjusted Akaike information criterion, Δ_i_*:* Akaike differences, *w_i_*: Akaike weights.

We compared the models using Δ_i_, which is the difference of AIC_c_ between a given model and the best model –the one with the lowest AIC_c_. We also calculated the AIC weights (w_i_) for each model, which represents the weight of the evidence that a certain model is the best model given the data and the set of candidate models [1]. The 95% confidence set of the best models was defined by summing the wi, from the largest to the smallest, until the sum is ≥ 0.95 [1].

**Referentes**

1. Burnham KP, Anderson DR (2002) Model Selection and Multimodel Inference: A Practical Information–Theoretic Approach. New York: Springer–Verlag. 2 ed.
