# Supplementary material for: Local and Landscape Factors Determining Occurrence of Phyllostomid Bats in Tropical Secondary Forests
Source: PLoS One. 2012 Apr 18;7(4):e35228. doi: 10.1371/journal.pone.0035228 (PMC3329449; doi:10.1371/journal.pone.0035228)
Supplement: Table S3 — Chiropterophylic and chiropterochoric species occurring in the Chamela-Cuixmala region. (DOC) [file pone.0035228.s004.doc]

Table S3. Chiropterophylic and chiropterochoric species occurring in the Chamela-Cuixmala region.

| **FAMILY** | **Chiropterophylic** | | **Chiropterochoric** | |
| --- | --- | --- | --- | --- |
| **Species** | **DF** | **RF** | **DF** | **RF** |
| **Anacardiaceae** |  |  |  |  |
| *Spondias purpurea* |  |  | X |  |
| **Apocynaceae** |  |  |  |  |
| *Stemmadenia donnell-smithii* |  |  | X |  |
| **Asparagaceae** |  |  |  |  |
| *Agave angustifolia* | X |  |  |  |
| *Agave colimana* | X |  |  |  |
| **Bignoniaceae** |  |  |  |  |
| *Crescentia alata* | X |  |  |  |
| *Cydista diversifolia* |  | X |  |  |
| **Boraginaceae** |  |  |  |  |
| *Cordia alliodora* | X | X |  |  |
| *Cordia gerascanthus* | X | X |  |  |
| **Bromeliaceae** |  |  |  |  |
| *Aechmea bracteata* |  |  |  | X |
| **Cactaceae** |  |  |  |  |
| *Pachycereus pecten-aboriginum* | X |  |  |  |
| *Stenocereus chrysocarpus* | X |  | X |  |
| *Stenocereus fricii** | X |  | X |  |
| *Stenocereus standleyi* | X |  | X |  |
| **Capparaceae** |  |  |  |  |
| *Capparis flexuosa* | X |  |  |  |
| *Crataeva tapia* | X |  |  |  |
| **Convolvulaceae** |  |  |  |  |
| *Ipomoea ampullacea* |  | X |  |  |
| *Ipomoea wolcottiana* | X |  |  |  |
| **Cucurbitaceae** |  |  |  |  |
| *Cucurbita argyrosperma* | X |  |  |  |
| **Fabaceae** |  |  |  |  |
| *Acacia farnesiana* | X |  |  |  |
| *Albizzia occidentalis* | X | X |  |  |
| *Bauhinia pauletia* | X |  |  |  |
| *Bauhinia ungulata* | X |  |  |  |
| *Calliandra formosa* | X |  |  |  |
| *Inga vera* | X |  |  |  |
| *Mucuna sloanei* |  | X |  |  |
| **Malvaceae** |  |  |  |  |
| *Ceiba aesculifolia* | X |  |  |  |
| *Ceiba grandiflora* | X |  |  |  |
| *Ceiba pentandra* |  | X |  |  |
| *Guazuma ulmifolia* |  |  | X |  |
| *Helicteres baruensis* | X |  |  |  |
| *Pseudobombax ellipticum* | X |  |  |  |

Table S3 (continue)

| **Family** | **Chiropterophylic** | | **Chiropterochoric** | |
| --- | --- | --- | --- | --- |
| **Species** | **DF** | **RF** | **DF** | **RF** |
| **Moraceae** |  |  |  |  |
| *Brosimum alicastrum* |  | X |  | X |
| *Chlorophora tinctoria* |  |  | X |  |
| *Ficus cotinifolia** |  |  | X |  |
| *Ficus insipida** |  |  |  | X |
| *Ficus obtusifolia** |  |  |  | X |
| *Ficus pertusa** |  |  |  | X |
| **Piperaceae** |  |  |  |  |
| *Piper arboreum** |  |  | X | X |
| *Piper hispidum* |  |  |  | X |
| **Sapindaceae** |  |  |  |  |
| *Sapindus saponaria* |  |  |  | X |
| **Sapotaceae** |  |  |  |  |
| *Pouteria campechiana* |  |  |  | X |
| **Solanaceae** |  |  |  |  |
| *Solanum erianthum* |  |  | X |  |
| *Solanum hazenii* |  |  | X |  |
| *Solanum diphyllum* |  |  |  | X |
| **Ulmaceae** |  |  |  |  |
| *Celtis iguanaea* |  |  |  | X |
| *Trema micrantha* |  |  | X |  |
| **Urticaceae** |  |  |  |  |
| *Urera caracasana* |  |  |  | X |

DF: dry forest, RF: riparian forest. * Seeds collected from bat feces during samplings.

We built the species list comparing the Neotropical bat/plant interactions database [1] against the checklist of vascular plants produced by Lott [2]. We also included the plant species collected during bat samplings (63 fecal samples with seeds), as well as the species *Stenocereus chrysocarpus, S. fricci* and *S. standleyi* based on Rojas-Martinez et al. [3]. Plant families follow the angiosperm phylogeny group classification (APG) [4]. Plant species names correspond to the nomenclature of Tropicos.org. Missouri Botanical Garden. 01 Apr 2010 (<http://www.tropicos.org/>).

**References**

1. Geiselman C, Mori SA, Lobova TA, Blanchard F (2002) onward Database of neotropical bat/plant interactions: <http://www.nybg.org/botany/tlobova/mori/batsplants/database/dbase_frameset.htm>
2. Lott EJ (2002) Lista anotada de la plantas vasculares de Chamela–Cuixmala. In: Noguera FA, Vega JH, García AN, Quesada M, eds. Historia Natural de Chamela. Instituto de Biología UNAM. México. pp 99–136.
3. Rojas-Martinez A, Valiente-Banuet A, Arizmendi MdelC, Alcantara-Eguren A, Arita HT (1999) Seasonal distribution of the long–nosed bat (*Leptonycteris curasoae*) in north america: does a generalized migration pattern really exist? Journal of Biogeography 26: 1065–1077.
4. APG III (2009) An update of the angiosperm phylogeny group classification for the orders and families of flowering plants: APG III. Botanical Journal of the Linnean Society 161: 105–121.
