# Supplementary material for: Local and Landscape Factors Determining Occurrence of Phyllostomid Bats in Tropical Secondary Forests
Source: PLoS One. 2012 Apr 18;7(4):e35228. doi: 10.1371/journal.pone.0035228 (PMC3329449; doi:10.1371/journal.pone.0035228)
Supplement: Methods S1 — Description of the image classification process. (DOC) [file pone.0035228.s005.doc]

## Description of the image classification process

The estimation of landscape metrics used as explanatory variables in the study were based on a classified image comprised of 4 ASTER, cloud free, satellite images, acquired for the Pacific coast of Mexico on December 28, 2005. This date is the most appropriate for the study as it represents an intermediate moment along the bat sampling period and corresponds to the dry season, when the highest differentiation between pastures, dry and riparian forest occurs [1,2]. The images were georeferenced to a map produced by Sanchez-Azofeifa et al. [2] on WGS84_UTM Zone 13 North. No atmospheric correction was applied to them.

For image classification, we employed the first three bands of the ASTER sensor (green, red and near infrared), as well as two other bands produced by the calculation of two indices: the normalized difference vegetation index (NDVI) and the single ratio (SR). These indexes are useful for the discrimination of successional stages of tropical dry forest [3,4]. Nominal spatial resolution of all bands was 15 m.

Image classification was based on a hierarchical classification procedure which classifies groups of pixels sequentially. During this process, we employed two algorithms for defining the group of pixels—an unsupervised classification and a supervised classification (ERDAS Imagine v.9.2, Leica Geosystems, Georgia, USA). Unsupervised classification was used for the definition of the following classes: riparian forest (RF, including both gallery forest located along large rivers and gallery forest located along temporary creeks), mangroves, oak forest, seasonal growing field (i.e. corn, tomato, hot pepper and watermelon), long term growing field (i.e. mango, papaya, coconut and citrus), bare soil (including dirt and paved roads) and water. Supervised classification was employed for the definition of dry forest initial successional stages (pastures and early successional stages) and dry forest advanced successional stages (DF, intermediate and late successional stages).

Supervised classification was based on training areas (surveyed during the 2004-2005 field work) spanning 200 pixels per class. Unsupervised classification was performed using as reference the high resolution imagery available on Google Earth ([http://earth.google.com](http://earth.google.com/)), as well as the classified image presented by Sanchez-Azofeifa et al. [2]. Finally, in order to eliminate the “noisy” results produced by wrongly classified pixels we used the neighborhood function available in ERDAS Imagine v.9.2. During this operation each pixel is analyzed and recoded, if necessary, based on the number and location of the pixels in the neighborhood.

For image validation we used 50 reference points per class collected in the field and on Google Earth ([http://earth.google.com](http://earth.google.com/)), which is considered an adequate number of points for the extension of our image and our number of classes [5]. The final accuracy of the classified image was estimated as 0.86 and 0.84 by the overall accuracy and Tau coefficient statistics, respectively. Analyses were performed with the software Image Analysis (ver 1.01) and Accuracy (ver. 1.0), created by T. de Camino-Beck at the Center for Earth Observation Sciences (CEOS), University of Alberta. Detailed information about statistical calculations can be found in Congalton [5] and Ma and Redmond [6].

## References

1. Kalacska M, Calvo-Alvarado GAJC, Sanchez-Azofeifa GA (2005) Calibration and assessment of seasonal changes in leaf area index of a tropical dry forest on different stages of succession. Tree Physiology 25: 733–744.
2. Sanchez-Azofeifa GA, Quesada M, Cuevas-Reyes P, Castillo A, Sanchez-Montoya G (2009) Land cover and conservation in the area of influence of the Chamela-Cuixmala Biosphere Reserve, Mexico. Forest Ecology and Management 258: 907–912.
3. Arroyo-Mora JP, Sánchez-Azofeifa GA, Rivard B, Calvo JC, Janzen DH (2005) Dynamics in landscape structure and composition for the Chorotega region, Costa Rica from 1960 to 2000. Agriculture, Ecosystems and Environment 106: 27–39.
4. Hartter J, Lucas C, Gaughan AE, Lizama LA (2008) Detecting tropical dry forest sucesión in a shifting cultivation mosaic of the Yucatán Peninsula, Mexico. Applied Geography 28: 134–149.
5. Congalton RG (1991) A review of assessing accuracy of classifications of remotely sensed data. Remote Sensing and Environment 37: 35–46.
6. Ma Z, Redmond RL (1995) Tau coefficients accuracy assessment of classification of remote sensing data. Photogrammetric Engineering and remote sensing 61: 435–439.
